# Voltammetric Determination of Ivabradine Hydrochloride Using Multiwalled Carbon Nanotubes Modified Electrode in Presence of Sodium Dodecyl Sulfate

**DOI:** 10.15171/apb.2017.019

**Published:** 2017-04-13

**Authors:** Ali Kamal Attia, Nisreen Farouk Abo-Talib, Marwa Hosny Tammam

**Affiliations:** National Organization for Drug Control and Research, P.O. Box 29, Cairo, Egypt.

**Keywords:** Multiwalled carbon nanotubes, Sodium dodecyl sulfate, Ivabradine hydrochloride, Voltammetry, Plasma

## Abstract

***Purpose:*** A new sensitive sensor was fabricated for the determination of ivabradine hydrochloride (IH) based on modification with multiwalled carbon nanotubes using sodium dodecyl sulfate as micellar medium to increase the sensitivity.

***Methods:*** The electrochemical behavior of IH was studied in Britton-Robinson buffer (pH: 2.0-11.0) using cyclic and differential pulse voltammetry.

***Results:*** The voltammetric response was linear over the range of 3.984 x 10^-6^-3.475 x 10^-5^ mol L^-1^. The limits of detection and quantification were found to be 5.160 x 10^-7^ and 1.720 x 10-6 mol L^-1^, respectively.

***Conclusion:*** This method is suitable for determination of IH in tablets and plasma.

## Introduction

Ivabradine HCl (IH) is used to reduce the heart rate through inhibition of the pacemaker current (*I*_f_)*.* IH is used in the treatment of heart failure, in sinus rhythm and angina pectoris when beta blockers are not responding.^1-3^

Several methods have been reported to determine IH such as spectrophotometric method,^4^ chromatographic methods,^4-11^ spectrofluorimetric method,^12^ and potentiometric method.^13^

The electroanalytical methods are simple, rapid, and in expensive techniques, they have great importance in environmental monitoring and pharmaceutical analysis.^14-20^

Carbon nanotubes (CNTs) have matchless geometrical, mechanical, electronic and chemical properties. Multi-walled carbon nanotubes (MWNTs) modified electrodes have plentiful characteristics compared with bare electrode according to their unrivaled properties. Nanoparticles increase the number of active sites and the rate of mass transport to the electrode surface.^21-24^

This study aims to determine IH at multiwalled carbon nanotubes modified carbon paste electrode (MWCNTCPE) utilizing voltammetric method based on the electrochemical oxidation of IH.

## Materials and Methods

### Apparatus

SP-150 (Biologic Science Instruments, France) was used for voltammetric experiments. The results were analyzed using EC-Lab software. Ag/AgCl (3.0 mol L^-1^ NaCl) reference electrode and a platinum wire counter electrode were purchased from BASi (USA), pH meter (JENWAY 3510, UK) was used to adjust buffer solutions. JSM-6700F scanning electron microscope (Japan Electro Company) was used to do scanning electron microscopy (SEM) experiments. FTIR-8400S spectrophotometer (Shimadzu, Japan) was used to obtain FTIR spectra of MWCNTCPE and MWCNTCPE/SDS. The charges of atoms of IH were calculated using Huckel^'^smethod (ChemBio 3D Ultra program).

### Materials and reagents

IH (98.5%) and Procoralan® tablets (5.39 mg of IH per tablet) were provided by Servier Egypt Industries Limited.

MWCNTs (6-13 nm in diameter and 2.5-20 μm in length; purity >98%), sodium dodecyl sulfate (SDS), Graphite and paraffin oil were supplied from Sigma-Aldrich. IH stock solution (1.0 x 10^-3^ mol L^-1^) and SDS solution (1.0 x 10^-2^ mol L^-1^) was prepared using deionized water.

Britton-Robinson (BR) buffer solutions (pH: 2.0-11.0) were prepared as mentioned before.^16^ Plasma was purchased from blood bank of VACSERA (Egypt).

### Working electrodes 

MWCNTCPE was made by mixing and stirring 1.0% (w/w) MWCNTs and 99% (w/w) graphite powder in ethyl ether to get good homogeneity, and then dry this mixture in air. The dried mixture was mixed with paraffin oil to obtain a uniformly wetted paste. The hole of the electrode was filled with paste and smoothed on a filter paper until a shiny appearance was obtained. A carbon paste electrode (CPE) was obtained using the same procedures without MWCNTs addition.

### Effect of SDS 

The cyclic voltammograms of IH (1.43 x 10^-4^ mol L^-1^) in BR buffer (pH 3) were recorded at MWCNTCPE upon successive addition of different volumes of SDS (1.0 x 10^-2^ mol L^-1^) to the voltammetric cell.

### Calibration curve of IH

Different volumes of IH solution (1.0 x 10^-3^ mol L^-1^) were added to 5 mL of BR buffer of pH 3.0. The solution was stirred for 5 s and the differential pulse voltammograms were done using scan rate of 10 mV s^-1^ at MWCNTCPE/SDS.

### Analysis of IH in tablets

Fifteen Procoralan tablets were grounded. Suitable amount needed to get IH solution of 1.0 x 10^-3^ mol L^-1^ was added to flask containing 60 mL deionized water, then dissolved by sonication for 15 min and the volume was completed to 100 mL with deionized water. The solution was filtered to remove the insoluble excipients. Standard addition method was performed to determine IH in dosage form.

### Analysis of IH in plasma

One mL of human plasma and 2 mL of acetonitrile were added to a series of 10 mL centrifuge tubes containing different volumes of IH (1.0 x 10^-3^ mol L^-1^), the mixture was centrifuged at 5000 rpm for 10 min to get rid of protein residues. 0.5 mL from the supernatant was transferred into voltammetric cell containing 4.5 mL of BR buffer (pH 3.0) and SDS solution (3.58 x 10^-4^ mol L^-1^). The procedures mentioned in calibration curve were done. The institutional board (NODCAR, Egypt) have agreed for testing with human subjects. Agreement was acquired from all contributors.

## Results and Discussion

### Voltammetric behavior of IH

**Figure 1:**
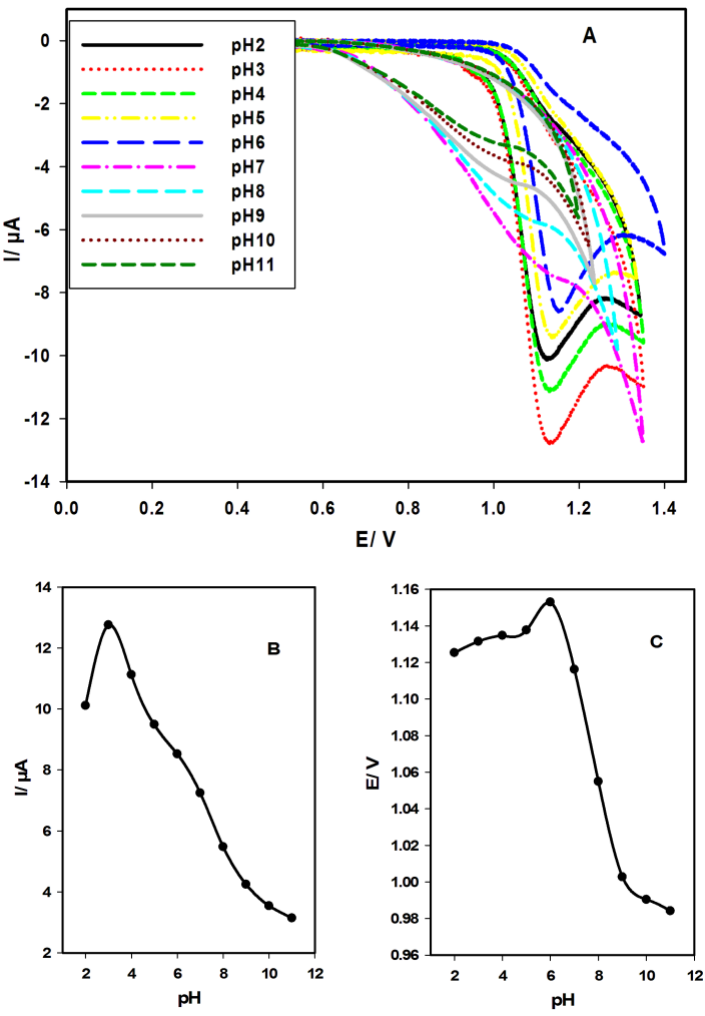
Cyclic voltammograms of the effect of solution pH on the oxidation of IH (1.43 x 10^-4^ mol L^-1^) at CPE using BR buffers (pH 2.0-11.0) at scan rate of 100 mV s^-1^ (A). Linear plots of anodic peak currents (B) and potentials (C) as a function of pH.

Figure 1A displays the cyclic voltammograms of IH (1.43 x 10^-4^ mol L^-1^) at CPE in BR buffer of different pH values. The forward scan shows anodic peak due to the oxidation process, while the reverse scan shows no peaks, indicating the irreversibility of the electrochemical process.

### Influence of pH

The electrochemical action of IH (1.43 x 10^-4^ mol L^-1^) was studied in different pH solutions (2.0-11.0) at CPE using cyclic voltammetry (CV) and scan rate of 100 mV s^-1^ as shown in Figure 1. Figure 1A shows that well defined and sharp anodic peaks in acidic medium (pH: 2.0-6.0) and broad peaks in neutral and basic medium (pH: 7.0-11.0). Figure 1 (A, B) shows that the anodic peak current presents the highest value at pH 3.0. Therefore, pH 3.0 was chosen to determine IH. Figure 1 (A, C) shows that the anodic peak potentials increases as pH increases up to pH 6.0, and decreases as pH increases up to pH 11.0.

**Figure 2:**
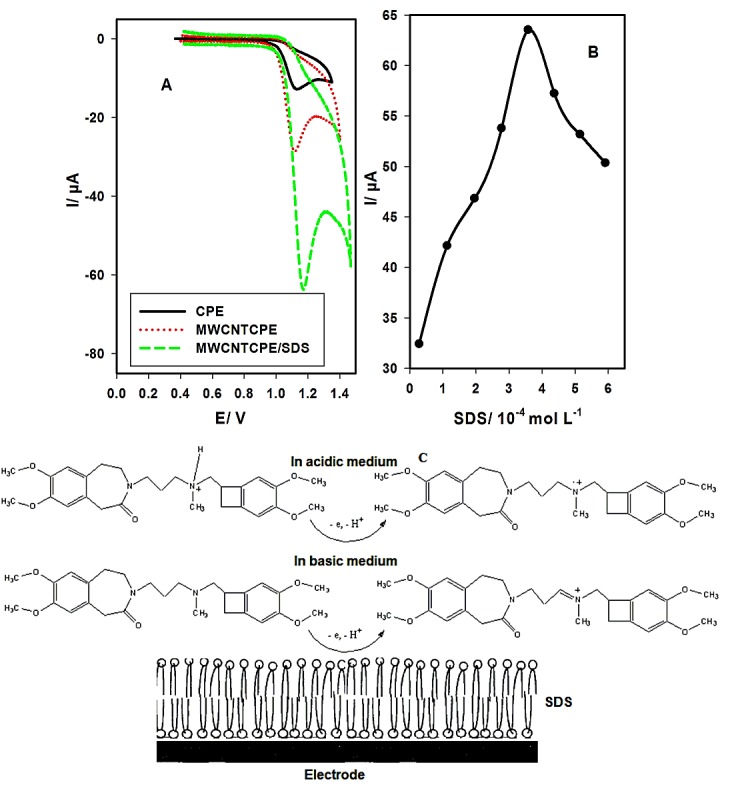
Cyclic voltammograms of IH (1.43 x 10^-4^ mol L^-1^) at CPE, MWCNTCPE and MWCNTCPE/SDS in BR buffer of pH 3.0 at scan rate of 100 mV s^-1^ (A), effect of SDS concentration on the anodic peak current of IH (B), the oxidation mechanism of IH at MWCNTCPE/SDS (C).

Figure 2A shows that the anodic peak currents (I) are 12.755 µA (at 1.131V), 28.641 µA (at 1.117 V) and 63.543 µA (at 1.171 V) at CPE, MWCNTCPE and MWCNTCPE/SDS, respectively. Graphite and multiwalled carbon nanotubes have hydrophobic surface and SDS molecule has hydrophobic tail giving hydrophobic interactions between the electrode surface and SDS molecules.^25,26^ SDS molecules were adsorbed on electrode surface to form negatively charged film which attract the positively charged drug and the drug concentration increases at the electrode surface leading to the increase of peak current.

Therefore, MWCNTCPE/SDS is the optimum electrode for the determination of IH according to its larger active surface area than CPE, and SDS works as micellar medium which increase the sensitivity and selectivity of IH. Electronic Supplementary Information 1 (ESI 1) shows the difference in the surface shape between CPE and MWCNTCPE according to their SEM. ESI 2 shows the FTIR spectra of MWCNTCPE and MWCNTCPE/SDS. MWCNTCPE does not show clear absorption peaks in its FTIR spectrum.^27,28^MWCNTCPE/SDS shows S-O-C vibration peaks at 850 cm^-1^ and 980 cm^-1^, C-O stretching vibration peak at 1040 cm^-1^, SO_2_ symmetric vibration peak at 1100 cm^-1^, CH_2_ scissoring at 1460 cm^-1^, CH_2_ stretching at 2890 (asymmetric) and 2830 cm^-1^ (symmetric), and a broad band between 3000 and 3650 cm^-1^ due to O-H stretching vibration.^29^

### Influence of SDS

Since IH is positively charged in acidic medium, SDS (as anionic surfactant) was used to enhance the peak current giving better sensitivity in the analysis of IH. Different volumes of SDS solution of concentrations varied from 2.85 x 10^-5^ to 5.91 x 10^-4^ mol L^-1^ were added to the electrolytic cell containing IH (1.43 x 10^-4^ mol L^-1^) in BR buffer (pH 3.0). Figure 2B shows that the peak current increases as the concentration of SDS increases up to 3.58 x 10^-4^ mol L^-1^ then after this concentration the peak current decreases as the concentration of SDS increases. Hence, the optimum SDS concentration was 3.58 x 10^-4^ mol L^-1^.

The mechanism of oxidation of IH is through the loss of one electron and one proton to form cation radical and cation in acidic and basic medium, respectively as shown in Figure 2C. The charges of atoms of IH were shown in ESI 3; N (amine) has the smallest negative value of -0.0586 (highest positive value) than those of the other atoms. Thus, it is the center of oxidation which loss one electron and its attached proton to form cation radical in acidic medium, while in basic medium this nitrogen atom loss one electron and the carbon atom C (18) which has the highest positive charge (0.0243) than those of the other carbon atoms loss one proton to form cation.

### Influence of scan rate

**Figure 3:**
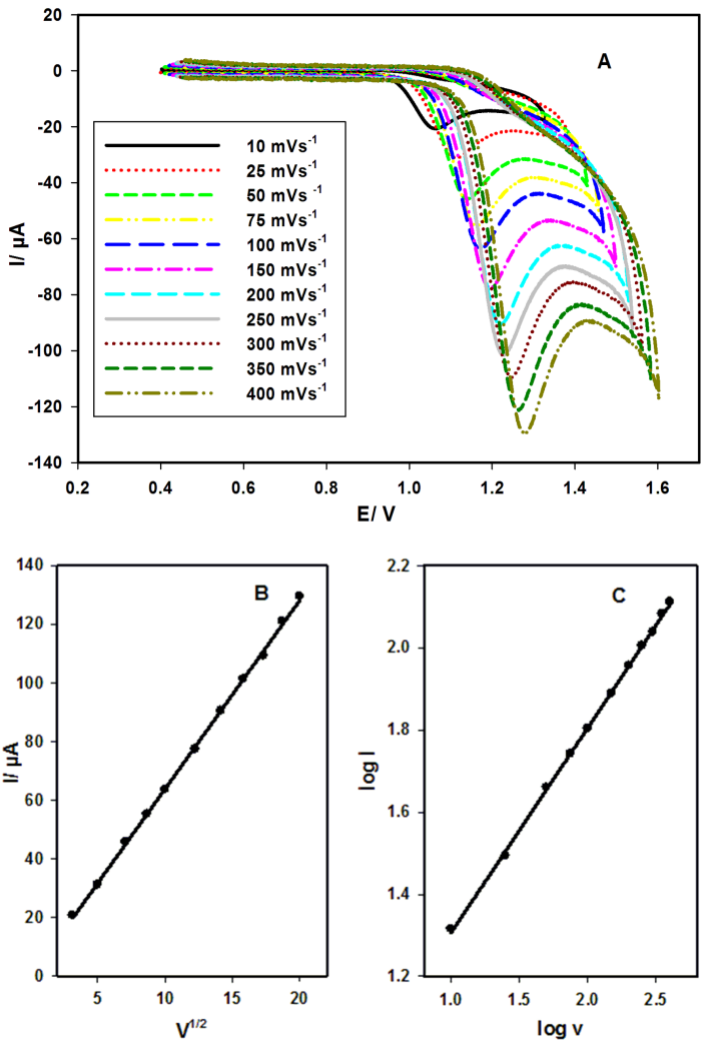
Effect of scan rate (10-400 mV s^-1^) on the anodic peak current of IH at MWCNTCPE/SDS, cyclic voltammograms (A), I vs. ʋ^1/2^ (B) and log I vs. log ʋ (C).

Figure 3 represents the oxidation of IH (1.43 x 10^-4^ mol L^-1^) in BR buffer (pH 3.0) as a function of scan rate (ʋ) (10-400 mV s^-1^) at MWCNTCPE/SDS. As ʋ increases, the peak current increases, and the peak potentials increases (Figure 3A). Figure 3B, 3C show linear relationships were found between the peak currents and ʋ^1/2^ and between the logarithms of the peak current and ʋ (log I = 0.80 + 0.48 log ʋ, R (Correlation coefficient) = 0.9997), the slope 0.48 is near to 0.50 (theoretical value) suggesting diffusion controlled process of the oxidation of IH.^30^

### Chronoamperometry study 

The diffusion coefficient of IH was determined in BR buffer (pH 3.0) at MWCNTCPE/SDS; the potential was set at 1.167 V. The diffusion coefficient of IH was determined using Cottrell equation: I = nFAC (D/πt)^1/2^where, I, n, F, C, D, and A are the current, the number of electrons (n = 1 for IH), Faraday constant (96480 C moL^-1^), analyte concentration (mol cm^-3^), the diffusion coefficient (cm^2^ s^-1^), and electroactive area of the working electrode, respectively.^31^ A was obtained using the diffusion coefficient of K_3_Fe (CN)_6_ which is equal to 7.6 x 10^-6^ cm^2^ s^-1^,^27^ and thus A was calculated to be 0.115 cm^2^.

**Figure 4:**
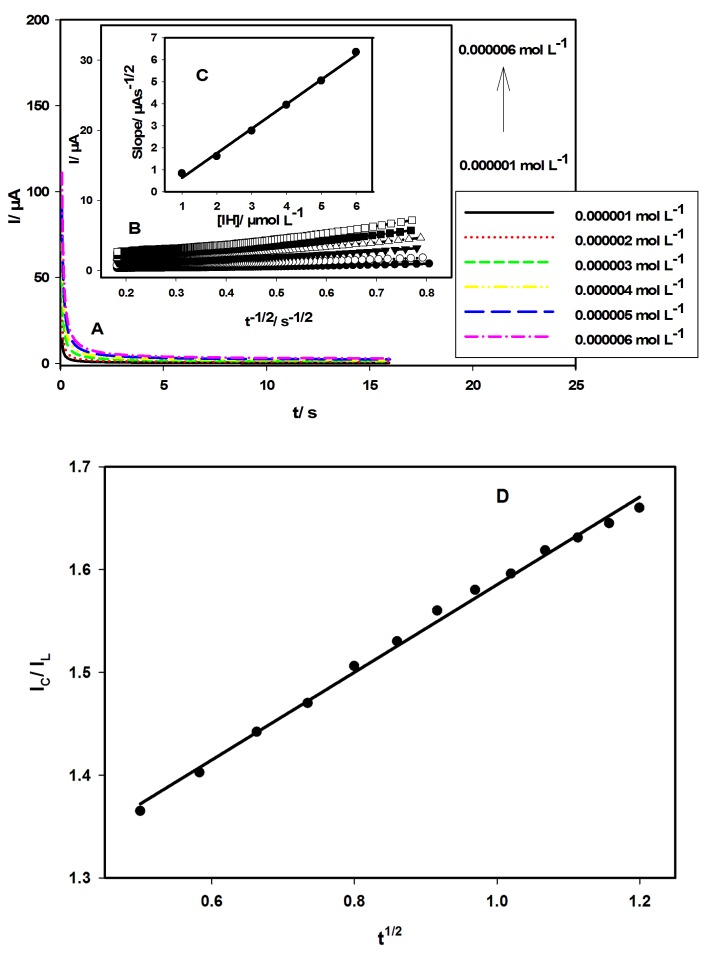
Chronoamperograms for IH at MWCNTCPE/SDS in BR buffer of pH 3.0 (A). Insets: I vs. t^1/2^from Cottrell’s plot obtained from chronoamperograms (B) and the plot of the slopes of the straight lines against IH concentrations (C). Plot of Ic/I_L_ vs. t^1/2^ in presence and absence of IH (3.0 x 10^-6^ mol L^-1^) in BR buffer of pH 3.0 (D).

Figure 4A represents the chronoamperograms of IH at MWCNTCPE/SDS in BR buffer of pH 3.0. It was shown that the chronoamperometric signal increases as the concentration of IH increases. It was found that 16 s is a sufficient electrolysis time to reach steady state. Figure 4B shows the linear relationships between I and t^-1/2^. The plot of the slopes of straight lines obtained in Figure 4B against the concentration of IH gives a straight line as shown in Figure 4C; the slope of this relation is used to calculate D based on Cottrell equation. D of IH was found to be 3.175 x 10^-5^ cm^2^ s^-1^.

The reaction rate constant (K) was determined using the following equation: I_C_/I_L_ = (πKCt)^1/2^ where I_C_ and I_L_ are the catalytic and limited currents in the presence and in the absence of IH, respectively.^32^ The value of K above equation was calculated from the slope of the plot of I_C_/I_L_ vs. t^1/2^for 3.0 x 10^-6^ mol L^-1^IH (Figure 4D), K was determined as 1.92 x 10^4^ moL^-1^ Ls^-1^.

### Determination of IH

**Figure 5:**
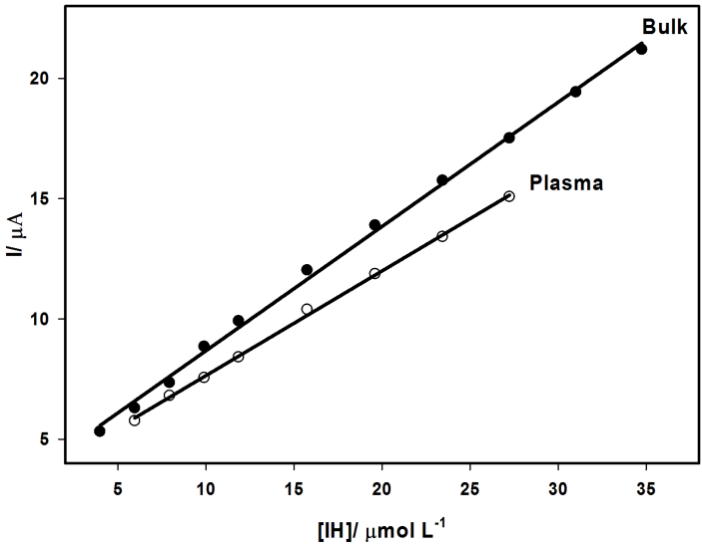
Calibration curves of IH in bulk and plasma using DPV at MWCNTCPE/SDS in BR buffer solution of pH 3.0, ʋ = 10 mV s^-1^.

Linear range, limits of detection (LOD) and quantification (LOQ) of IH were obtained using differential pulse voltammetry (DPV) at the MWCNTCPE/SDS. Figure 5 depicts the calibration curve of IH (3.984 x 10^-6^ - 3.475 x 10^-5^mol L^-1^), I (µA) = 3.51 + 0.52 C (µmol L^-1^), R = 0.9994. LOD and LOQ were found to be 5.160 x 10^-7^ and 1.720 x 10^-6^ mol L^-1^, respectively.

Table 1 shows comparison between the proposed method and some reported methods used for analysis of IH. The proposed DPV method is more sensitive than these methods.

Statistical comparison between the results obtained by proposed voltammetric method and reported method,^11^ was performed using t- test and F-ratio.^33^ There is no significance difference between them as shown in Table 1.

Table 2 shows the repeatability of the proposed method using different concentrations of IH which each of them was measured three times a day for three successive days.

The proposed method shows good repeatability as shown in Table 2.

The reproducibility of the proposed method was done by two different analysts using the same procedures for analysis of IH (9.9 x 10^-6^ mol L^-1^). The recovery values were 99.65% and 100.48% for the first and the second analyst, respectively. The relative standard deviations of three replicate measurements were 0.61% and 0.74% for the first and the second analyst, respectively, suggesting good agreement of results.

**Table 1:** Comparison between the proposed DPV method and some other reported methods used to determine IH. Statistical analysis of the proposed method and the reported HPLC method for determination of IH.^11^

**Method**	**Linear range**	**Reference**
DPV (mol L^-1^) (µg mL^-1^)	3.984 x 10^-6^- 3.475 x 10^-5^ (2.012 - 17.550)	This work
Spectrophotometry (µg mL^-1^)	4.2 - 31.6	[4]
Chromatography (µg mL^-1^)	4.2 - 31.6	[4]
	70.69 - 131.29	[11]
Potentiometry (mol L^-1^)	1.0 x 10^-5^ - 1.0 x 10^-2^	[13]
**Statistical term**	**Proposed method**	**Reported method** ^11^
%Mean recovery	100.316	100.852
SD	1.593	1.450
Variance	2.537	2.163
n	5	5
*t*-test (2.306)^*^	0.553	
*F*-ratio (6.39)^*^	1.173	

^*^Figures in parenthesis are the theoretical values of t and F at conﬁdence limit 95%.

### Interference study

Lactose, microcrystalline cellulose, titanium dioxide and magnesium stearate are used as excipients in pharmaceutical industry. Interference studies were performed prior to analysis of IH in dosage forms using 1.0 x 10^-5^ mol L^-1^ and1.0 x 10^-4^ mol L^-1^ of IH and all excipients, respectively. The presence of excipient not affect drug estimate.

### Analysis of IH in tablets

Standard addition method was applied for analysis of IH in Procoralan tablets without any extraction steps prior to the analysis. The results showed that interference from the matrix was negligible (Table 2). IH can be determined in pharmaceutical formulation within the linear range (3.984 x 10^-6^ - 3.475 x 10^-5^ mol L^-1^).

### Analysis of IH in plasma

DPV method was successfully used to determine IH in spiked human plasma over the range of 5.964 x 10^-6^-2.723 x 10^-5^ mol L^-1^ (Figure 5) obeying analytical equation: I (µA) = 3.29 + 0.43 C (µmol L^-1^), R = 0.9991. LOD and LOQ were 1.15 x 10^-6^ and 3.82 x 10^-6^ mol L^-1^, respectively. The recovery values were in the range of 99.16-102.32%. The relative standard deviation was 0.996%.

**Table 2:** Precision data for the proposed method. Determination of IH in Procoralan tablets by applying standard addition method.

**Concentration** **(mol L**^-1^**)**	**Intra-day precision**		**%Mean** **Recovery±SD**	**%RSD**
	**Amount found** ^(a)^	**%Recovery** ^(a)^		
7.936 x 10^-6^	7.938 x 10^-6^	100.025	99.854±0.372	0.372
1.574 x 10^-5^	1.565 x 10^-5^	99.428		
2.723 x 10^-5^	2.726 x 10^-5^	100.110		
**Concentration** **(mol L**^-1^**)**	**Inter-day precision**		**%Mean** **Recovery±SD**	**%RSD**
	**Amount Found** ^(a)^	**%Recovery** ^(a)^		
7.936 x 10^-6^	7.931 x 10^-6^	99.937	99.630±0.403	0.404
1.574 x 10^-5^	1.561 x 10^-5^	99.174		
2.723 x 10^-5^	2.717 x 10^-5^	99.780		
**Dosage form**	**IH (mol L**^-1^**)** **Taken**	**IH (mol L**^-1^**)**		**Recovery (%)**
		**Added**	**Found**	
Procoralan	5.964 x 10^-6^	1.984 x 10^-6^	7.880 x 10^-6^	99.144
		3.964 x 10^-6^	9.898 x 10^-6^	99.698
		5.940 x 10^-6^	11.95 x 10^-6^	100.386
		7.912 x 10^-6^	13.78 x 10^-6^	99.308
	Mean recovery ± RSD*%			99.634±0.553

^a^Mean of three different samples for each concentration. SD: Standard deviation of three different determinations RSD: Relative standard deviation

## Conclusion

The proposed method used MWCNTs and SDS based on their properties for the quantitative determination of IH in bulk, tablets and plasma. The sensor sensitivity and selectivity were enhanced using MWCNTs and SDS in comparison with CPE. The proposed DPV method is not time consuming method, there is no extraction stage. It can be used for quality control of IH.

## Acknowledgments

The authors would like to express their gratitude to the National Organization for Drug Control and Research (NODCAR, Egypt) for providing instruments and the means necessary to accomplish this work.

## Ethical Issues

Not applicable.

## Conflict of Interest

Authors declare no conflict of interest in this study.
